# Hypoxia Induced ER Stress Response as an Adaptive Mechanism in Cancer

**DOI:** 10.3390/ijms20030749

**Published:** 2019-02-11

**Authors:** Sandhya Chipurupalli, Elango Kannan, Vinay Tergaonkar, Richard D’Andrea, Nirmal Robinson

**Affiliations:** 1 Department of Pharmacology, JSS College of Pharmacy, Ooty 643001, India; sandhyach2304@gmail.com (S.C.); elangojss@gmail.com (E.K.); 2 Centre for Cancer Biology, University of South Australia and SA Patholology, Adelaide 5001, Australia; vinayt@imcb.a-star.edu.sg (V.T.); Richard.DAndrea@unisa.edu.au (R.D.); 3 Laboratory of NF-κB Signaling, Institute of Molecular and Cell Biology (IMCB), Proteos 138673, Singapore; 4 Cologne Excellence Cluster on Cellular Stress Responses in Aging-Associated Diseases (CECAD), University of Cologne, 50931 Cologne, Germany

**Keywords:** hypoxia, endoplasmic reticulum, UPR, cancer, HIF-1, stress-response

## Abstract

It is evident that regions within tumors are deprived of oxygen, which makes the microenvironment hypoxic. Cancer cells experiencing hypoxia undergo metabolic alterations and cytoprotective adaptive mechanisms to survive such stringent conditions. While such mechanisms provide potential therapeutic targets, the mechanisms by which hypoxia regulates adaptive responses—such as ER stress response, unfolded protein response (UPR), anti-oxidative responses, and autophagy—remain elusive. In this review, we summarize the complex interplay between hypoxia and the ER stress signaling pathways that are activated in the hypoxic microenvironment of the tumors.

## 1. Introduction

Cancers are often challenged by their typical microenvironment, termed tumor microenvironment (TME), which has a major impact on cancer progression. The understanding of TME is gaining importance for identifying ways to control cancer cells. TME can be subdivided into a chemical microenvironment and a cellular microenvironment, wherein the former encompasses pH, pO_2_, and a concentration of metabolites such as glucose, glutamate, and lactate [1]. The tumor cellular microenvironment is comprised of blood vessels, immune suppressor cells, fibroblasts, lymphocytes, bone marrow-derived inflammatory cells, extracellular matrix (ECM), and stromal cells, which influence the growth of cancerous cells [2,3]. TME has been shown to regulate cell growth and determine the potential of metastasis, and it also impacts the therapeutic outcome [4]. The stromal cells are not malignant, but their role in supporting the growth of cancer cells is found to be very important for tumor progression. Malformed tumor vasculature contributes to acidosis and tumor hypoxia and increases the interstitial fluid pressure. Reciprocally, the tumor responds by expressing a unique repertoire of genes that alter cellular growth, invasion, and ultimately metastasis [4]. 

One of the key impediments for therapies that prevent tumor progression is the hypoxic environment in which the cancer cells thrive [5,6,7]. Tumor hypoxia arises as a result of imbalance between oxygen demand (high metabolic demand) and its supply to the tissue, which is associated with poor structural and functional vasculature. This correlates with the aggressive phenotype of tumors and their resistance to conventional therapies [4]. Hypoxic cells in a tumor mass undergo a metabolic shift from oxidative phosphorylation to rapid glycolysis and also accumulate free radicals, resulting in the development of metabolic stress. In order to cope with such stress and maintain cellular homeostasis, hypoxic cells activate adaptive responses, such as alternate metabolic pathways, autophagy, and anti-oxidative responses [8]. Additionally, hypoxia is also known to stress the endoplasmic reticulum due to the accumulation of misfolded proteins, which activates an unfolded protein response (UPR). These adaptive responses are pro-survival mechanisms that are not induced in normal healthy tissues, hence these pathways can provide potential targets for anti-cancer therapy. In this review, we focus on the role of UPR in hypoxia-induced tumor progression and the molecular links between hypoxia and UPR.

## 2. Hypoxia in Tumor Progression

Solid tumors are often exposed to different gradient levels of oxygen, and as they develop, some regions receive low oxygen levels, leading to the generation of hypoxic regions because of the extreme energy demands of rapidly dividing cells. A hallmark of cancer is the induction of angiogenesis, which provides a vascular network to supply both oxygen and nutrients [9]. Tumor vasculature is disorganized, irregular, and is less efficient in the transport of oxygen and other nutrients. Thus, tumor cell exposure to hypoxia correlates with the advanced stages of malignancy, which ultimately results in resistance to both chemotherapy and radiotherapy. A major mechanism mediating adaptive responses to hypoxia is the transcriptional program activated by hypoxia-inducible factor 1 (HIF-1) [10]. While it is well established that hypoxia can trigger apoptosis or necrosis, it can also prevent cell death by stimulating adaptive responses that promote cell proliferation, survival, and angiogenesis, thus contributing to cancer progression [11]. One of the vital pathways mediating this response is allied with the activation of HIF-1, which was first described by Wang and Semenza in 1995 [12]. It is evident that activation of HIF-1 activates pro-survival as well as pro-death decisions under hypoxia [11,13]. Therefore, it is vital to understand the decision-making processes that regulate cell death, adaptation, and resistance to therapy, and the tumor properties that impact these. 

Expression of HIF-1 and associated pathways is linked to the development and pathophysiological aspects of many human diseases [14]. Likewise, intratumoral hypoxia leads to the sustained expression of HIF-1α, resulting in genetic instability and phenotypic diversity in solid tumors including prostate, breast, bladder, brain, colon, ovarian, and pancreatic tumors, but it is not expressed in the surrounding normal tissue [15,16]. Within the solid tumors, the interior mass turns hypoxic during its quick expansion until sufficient blood vessels are formed by the tumors. Thus, the hypoxic conditions within the tumor can lead to enhanced stability and activation of HIF-1α. Immunohistochemical studies have shown appreciable expression of HIF-1α in some benign tumors. However, it is enhanced in primary malignant tumors and further increased in metastatic tumors, while being absent in normal healthy tissues [16,17]. In addition to this, overexpression of HIF-1α has been detected in 69% of the metastatic breast cancers. However, the significant role of HIF-1α on the metastatic potential and cancer progression is studied only in gastric cancer [18] but is yet to be investigated in other metastatic cancers. Furthermore, a noticeable frequency of genetic alterations in tumor cells is associated with the enhanced expression of HIF-1α [19,20]. In clinical studies, expression of HIF-1α has been suggested as a marker of a highly aggressive disease associated with poor prognosis and treatment failure in numerous cancers [21,22,23,24]. HIF-1α has been used as a marker to identify lymph node-negative breast cancer patients who are at increased risk of treatment failure and death even though their tumors are histopathologically classified as low grade [23]. Similarly, in oropharyngeal cancers, patients with an increased expression of HIF-1α in more than 10% of their tumor cells were found to be thrice more likely to fail radiation therapy [24]. HIF-1α levels are also increased in tumors with activated PI3K/AKT signaling; this mechanism is well understood in prostate cancer cells where the inactivation of *PTEN* facilitates the HIF-1-mediated gene expression, leading to increased tumor vascularity and growth compared to cells expressing PTEN [25]. However, it is important to note that the correlation between the overexpression of HIF-1α, resistance, and poor prognosis is not universal. For example, in a lung cancer study, the overexpression of HIF-1α was found to be correlated with apoptosis and patient survival; however, this finding was not established in another study [21,22,23,24,25]. In acute myeloid leukaemia (AML) and other hematologic malignancies, the situation is also complex. While there is good evidence supporting the role of HIF in adaptation to hypoxia by primitive hematopoietic stem and progenitor cells, there is still an incomplete understanding of the role of HIF-1α (and HIF-2α) in AML development, and the role of HIF may depend on a number of parameters in this heterogeneous disease setting [26].

## 3. Regulation of HIF-1

**Figure 1:**
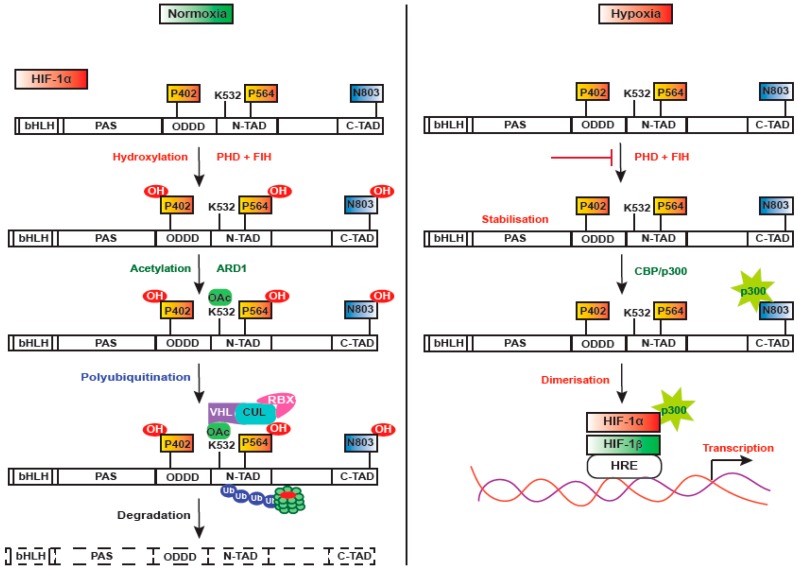
Regulation of HIF-1alpha under normoxia and hypoxia. In normoxia, P^402^ and P^564^ proline residues and N^803^ residue are hydroxylated by prolyl hydroxylases (PHDs) and factor inhibiting HIF-1 (FIH-1), respectively, in an O_2_-dependent manner, followed by ADP-ribosylation factor domain protein-1 (ARD1) dependent Acetylation of K^532^ lysine residue. Hydroxylated HIF-1 then binds to VHL E3 ubiquitin ligase complex, leading to its proteasomal degradation. Hydroxylated N^803^ blocks the recruitment of the CBP/P300 transcriptional coactivator, whereas during hypoxia, PHDs and FIH-1 are blocked, thus inhibiting the hydroxylation of proline and asparagine residues. Lack of hydroxylation prevents the binding of VHL, thus stabilizing HIF-1alpha, which translocates into the nucleus, allowing the recruitment of CBP/P300 and gene transcription.

**Figure 2:**
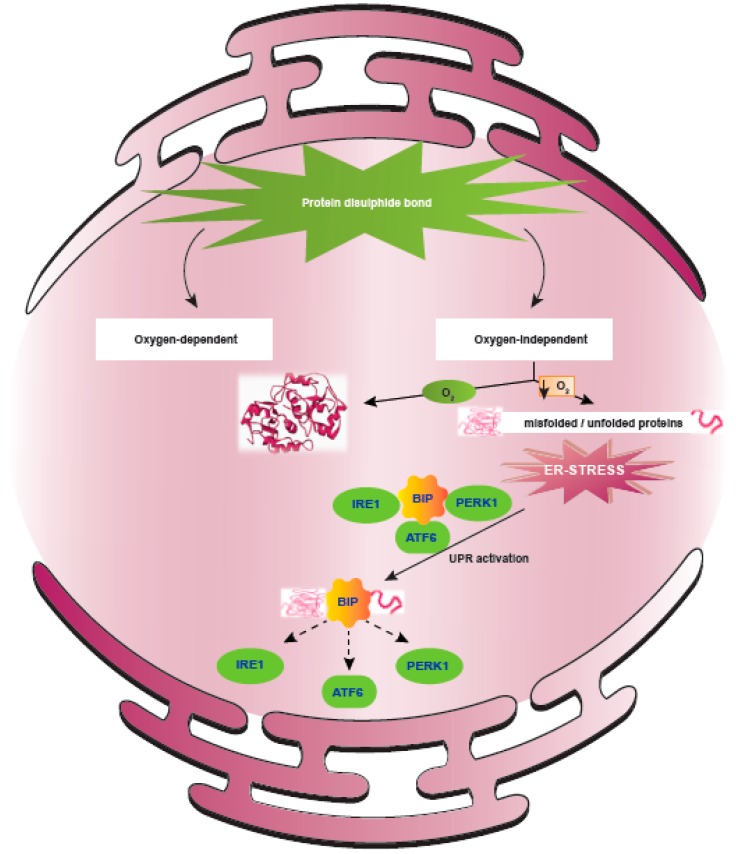
Hypoxia induced ER stress. Endoplasmic reticulum is the central organelle responsible for protein translational modifications, wherein the formation of protein disulphide bond is mediated by protein disulphide isomerase (PDI), an ER chaperone. During protein synthesis, protein disulphide bond is independent of oxygen, but protein folding is dependent on oxygen. Hence, in solid tumors, decreased availability of oxygen (hypoxic regions/fraction) causes perturbations in protein folding and results in the accumulation of misfolded/unfolded proteins. These changes disturb the ER proteostasis, leading to “ER Stress” and activation of unfolded protein response (UPR) as an adaptive mechanism.

HIF-1 is a heterodimer consisting of two functional subunits—HIF-1α and HIF-1β (ARNT—aryl hydrocarbon receptor nuclear translocator) [12]. HIF-1α has an oxygen domain, which is highly regulated by oxygen concentration and has a short half-life (approximately 5 min) [23,27]. It controls the expression of a variety of genes that play crucial roles in acute and chronic adaptation to oxygen deficiency, such as erythropoiesis, glycolysis, angiogenesis, inhibition of apoptosis, and cell differentiation [28,29,30,31]. HIF-1α undergoes multiple modes of post-translational modifications during normoxia, as it is expeditiously downregulated in an oxygen-dependent manner. In normoxia (Figure 1), HIF-1α is rapidly degraded by the proline hydroxylases-pVHL-proteasome system, but during hypoxia, HIF-1α is stabilized and translocated into the nucleus, where it dimerizes with HIF-1β and forms a transcriptionally active HIF complex [32,33]. The proteostasis of HIF-1α is critically regulated by ubiquitination mediated by the protein von Hippel-Lindau (pVHL). It directly binds to the oxygen degradation domain of HIF-1α facilitated by prolyl-4-hydroxylase (PHD), which hydroxylates two specific proline residues—Pro402 and Pro564—in humans [34,35]. VHL recruits a ubiquitin ligase protein complex consisting of elongin B, elongin C, and cullin, which ultimately results in ubiquitination and degradation of HIF-1α by the 26S proteasome [36,37]. PHDs are dioxygenases that require molecular oxygen, Fe^2+^, and 2-oxoglutarate as substrates [38]. Among the four identified PHDs that have distinct functions, PHD2 is found to be the critical oxygen sensor that maintains steady-state levels of HIF-1α under normoxia. Thus, PHDs provide HIF-dependent auto-regulatory mechanisms driven by oxygen concentrations (Figure 2). 

The stability of HIF-1α is also known to be regulated by VHL-independent mechanisms. MDM-2 mediated ubiquitination and proteasomal degradation of HIF-1α has been reported, wherein MDM2 is the E3 ligase that induces the hypoxic degradation of HIF1α. Moreover, the action of MDM2 on HIF1α under hypoxia occurs in the cytoplasm and is controlled by the PTEN-PI3K-AKT signaling axis [39]. Heat shock protein 90 (Hsp-90)-dependent degradation of HIF-1α has also been reported, in which HSP90 directly interacts with HIF-1α, causing a conformational change in response to dimerization with HIF-1β [40,41]. The PI3K/AKT pathway and the mammalian target of rapamycin (mTOR)-dependent phosphorylation of eukaryotic initiation factor 4E (eIF4E) under normoxic conditions have also been shown to increase HIF-1α. However, under hypoxia, mTOR increases the levels of HIF-1α by a mechanism that does not involve eIF4E [42]. Additionally, asparagine hydroxylase decreases the binding of the p300 transcriptional co-activator, thus reducing the activity of HIF1α [43,44,45]. Given the multiple mechanisms that regulate HIF1α, it is likely that alternate mechanisms will be identified across different cancer types, and hence further studies are needed to explore approaches for targeting HIF1 in specific cancer settings.

## 4. Stress Responses Regulated by HIF-1 in Response to Hypoxia

The hypoxic microenvironment in solid tumors is a result of rapid consumption of available oxygen within 70-150µm of tumor vasculature by rapidly proliferating cells, thus restraining the amount of oxygen available to diffuse into tumor tissue. In order to support uninterrupted growth and proliferation in the hypoxic environment, cancer cells have evolved to survive and multiply by altering their metabolism [3,46]. In hypoxic cells, mitochondrial oxidative phosphorylation is downregulated and taken over by aerobic glycolysis. This metabolic shift is termed as the “Warburg effect”, which enables rapid generation of ATP at the expense of large amounts of glucose [47]. The breakdown of glucose enhances lactic acid levels, which may further cause acidosis [11]. Hypoxia combined with acidosis induces adaptive stress responses in cancer cells, which interestingly promotes aggressive cancer phenotypes with the enhanced ability to invade and metastasize. It is becoming increasingly evident that hypoxia exerts significant effects on cellular metabolism via HIF-1α and is found to be the common link between hypoxia, metabolic adaptation, and tumor progression [48]. HIF1α critically regulates the switch to glycolysis by activating the transcription of genes encoding for glycolytic enzymes, such as Lactate dehydrogenase A (LDHA), phosphoglycerate kinase 1 (PGK-1), hexokinase-1 (HK1), and the glucose transporter, glucose transporter 1(GLUT1) [49]. During hypoxia, glucose entry to the TCA cycle is prevented by HIF-1α, which indirectly regulates pyruvate dehydrogenase (PDH) enzyme activity through pyruvate dehydrogenase kinase 1 (PDK1). PDK1 is upregulated during hypoxia, which further inhibits PDH and thereby prevents the conversion of acetyl coenzyme A from pyruvate [48]. 

It is also observed that NAD+ dependent deacetylase Sirtuin 1 (SIRT1) plays a role in maintaining the redox balance. SIRT1 and HIF-1α are coregulated, where the former acts as a redox sensor and the latter acts as an oxygen sensor [50]. Lim et al. [50] reported that SIRT1 modulates cellular responses to hypoxia by deacetylating HIF-1α. In normoxic cells, SIRT1 inactivates HIF-1α by deacetylating the Lys674 residue, thus preventing p300 recruitment and repressing HIF-1 target genes. However, during hypoxia, SIRT1 is downregulated, which allows acetylation of HIF-1α at Lys674 by p300/CBP-associated factor (PCAF) and thus the activation of HIF-1α [50]. These results suggest that the crosstalk between oxygen- and redox-responsive signaling occurs through the interaction between SIRT1 and HIF-1α.

The redox signaling necessary for various cellular functions is mediated by reactive oxygen species (ROS). In normal cells, increased levels of ROS can cause cell death. However, a range of adaptive changes reported in cancer cells allow modulation of redox homeostasis such that tumor cells survive in the presence of elevated ROS. Mitochondria are one of the major sources of intracellular ROS [51,52]. Electron transport chain complex I and III are the important sites of mitochondria, which have significant roles in redox signaling [51,53]. Several factors augment the production and release of mitochondrial ROS (mROS), among which tumor hypoxia is an important driving factor [54,55,56,57]. In most cancers, ROS activates pro-tumorigenic signaling pathways, such as PI3K/AKT, MAPK/ERK, and HIF-1α, and this is often associated with the inactivation of negative regulators for these pathways, such as PTEN, MAPK phosphatase, and PHD-2, which also promote proliferation, survival, and metastasis. In addition to tumor hypoxia, downregulation of antioxidant systems in breast cancer cells further potentiates ROS production through the loss of SIRT-3 function induced by the accumulation of mROS and the stabilization of HIF-1α [58]. Nuclear factor E2-related factor 2 (NRF2) is another transcription factor that plays a critical role in redox homeostasis by transcriptionally activating antioxidative responsive genes. Under physiological conditions, NRF2 is present in low levels, as it is ubiquitinated and degraded in the proteosome by kelch-like ECH associated protein 1 (KEAP1)-E3 ubiquitin ligase complex. However, during oxidative stress, ROS disrupt the KEAP1-NRF2 interaction, resulting in the accumulation and activation of NRF2. A large body of evidences suggest that NRF2 accumulates and is transactivated in various cancers [59].

Finally, autophagy plays a key role as an adaptive response in tumor cells associated with high levels of stress pathway activation and allows tumors to maintain metabolic homeostasis [60,61]. Autophagy is an important process in healthy cells that is responsible for mitochondrial turnover and removal of damaged mitochondria. Impaired autophagy is implicated in tumor-initiation through defective mitophagy and de-regulated ROS. The precise role of autophagy in cancer is still a subject of intense debate; autophagy has been shown to support tumor cell survival and to lead to death-promoting signals in response to microenvironmental stress factors [62]. Thus, the role of autophagy in cancer remains complex and accumulating evidence shows that autophagy is critical in tumor responses to therapy by leading to chemotherapeutic resistance [62]. For instance, it has been shown that the inhibition of glycolysis by 2-deoxyglucose results in ER stress, which causes active conversion of LC3-I/II and confers autophagy-dependent cell survival [63]. On the other hand, autophagy can also induce caspase-dependent cell death in cancer cells. In 2010, Norman et al. showed that cleavage of autophagic proteins is observed when cells switch to apoptosis over autophagy. For example, it has been revealed that the autophagic protein, Atg5, is cleaved by calpain, resulting in caspase activation [64]. In response to metabolic stress in cancer cells, the balance between cell growth and autophagy is regulated primarily by the mammalian target of rapamycin (mTOR) [65]. mTOR is inhibited by the product of the *BCL2/adenovirus E1B 19 kDa protein-interacting protein 3 (BNIP3)* gene, which is regulated in response to hypoxia [66]. It has also been reported that induction of hypoxia induces mitochondrial autophagy, which is regulated by HIF-1-dependent expression of BNIP3 [67]. Nevertheless, an intriguing question remains regarding the function of the cell death promoting protein (BNIP3) induced under hypoxic conditions, where HIF-1α is shown to promote cell survival. Conversely, BNIP3 expression is found to reach maximum during severe hypoxia and is observed close to the necrotic tumor areas. Indeed, Bellot et al. also showed that hypoxia-induced autophagy is mediated by HIF-1α –induced BNIP3, which was demonstrated to promote tumor cells survival and progression [68]. In addition, mTOR has been reported to be regulated by hypoxia via *Regulated in Development and DNA Damage 1* (REDD1) [69], which also regulates mTOR and autophagy in response to ER stress [70]. Despite these insights, much remains to be elucidated with regard to the signaling pathways that lead to ER stress-mediated autophagy in hypoxic tumors. A much more complete understanding of the molecular mechanisms at play between hypoxia-induced ER stress and autophagy will provide insights for development of new anti-cancer therapies. 

## 5. ER Stress Response Regulated upon Hypoxia

The ER is the largest organelle besides the nucleus and has an extensive membranous network of tubules, vesicles, and a sac that surrounds the nucleus and expands to the cytosol [71,72]. The ER is a compartment enriched in calcium, which orchestrates protein folding, assembly, and is also a site for lipid and sterol biosynthesis [73,74]. Inside the ER, posttranslational modifications including disulfide bond formation and N-linked glycosylation play major roles in the protein folding and assembly. Properly folded proteins in the lumen of the ER are transported out, while unfolded/misfolded proteins are retained in the ER and eventually degraded [73]. However, a number of biochemical, physiological, and pathological stimuli—which can cause oxidative stress, ER calcium depletion, nutrient deprivation, altered glycosylation, DNA damage, or energy perturbations—can disrupt the protein folding and subsequently cause an accumulation of unfolded/misfolded proteins in the ER. This is a condition referred to as “Endoplasmic Reticulum stress–ER stress” [74,75,76,77,78,79]. The cells respond by modulating signaling pathways that activate a transcriptional program to alleviate ER stress, termed as the unfolded protein response (UPR) [74,78].

**Figure 3:**
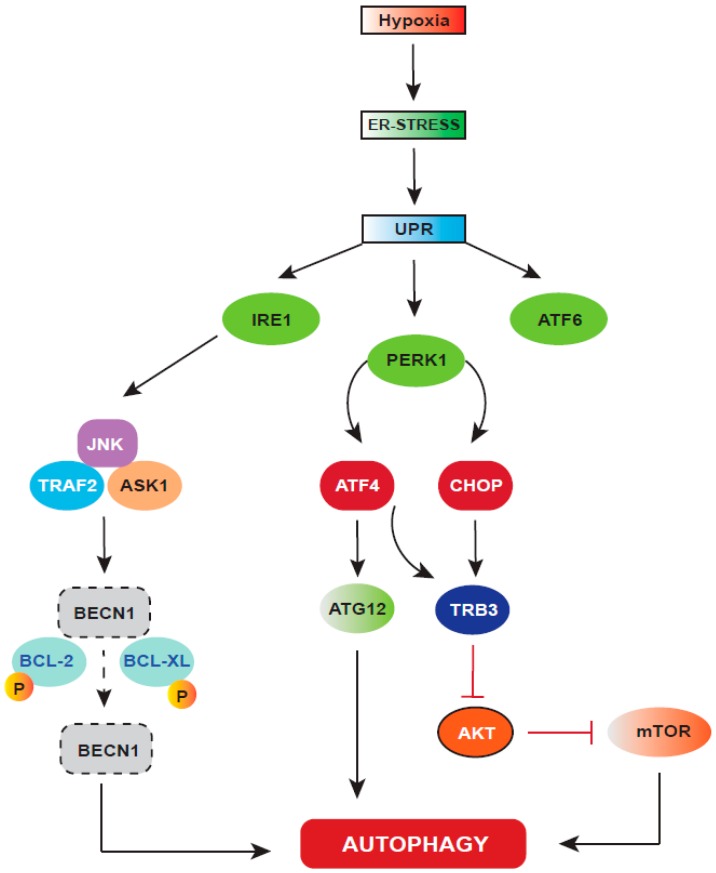
Hypoxic stress induced unfolded protein response (UPR) and autophagy. Hypoxia causes perturbations in the ER activity, resulting in UPR activation. UPR has three signaling arms, which play differentially in different tumors and also in different stages of tumor development and progression. UPR-activation induces autophagy through IRE1 and PERK signaling. Activated IRE1 phosphorylates JNK by recruiting TRAF2 and ASK1, which phosphorylate two autophagy inhibitor proteins, BCL-2 and BCL-XL, leading to their dissociation from BECN1, the key autophagy inducer. In addition to this, activated PERK drives the downstream expression of both ATF4 and CHOP, where ATF4 drives the expression of ATG12, and in combination with CHOP, ATF4 regulates the expression of TRB3, which blocks mTOR by inhibiting AKT, thus inducing autophagy.

Cancer cells experiencing hypoxia display extensive protein modification in the ER, which leads to the accumulation of misfolded/unfolded proteins. During protein synthesis in the ER, formation of disulphide bonds is the major post-translational modification step, catalyzed by the protein disulphide isomerase family of enzymes. It is evident from recent studies that disulphide bond formation during protein synthesis is independent of oxygen, in contrast to that which occurs during post-translational protein folding/isomerization, which is oxygen-dependent [80]. Thus, under hypoxic conditions, there is an accumulation of misfolded/unfolded proteins in the ER, and this triggers UPR, which promotes survival of cancer cells as well as mediates resistance to available anti-cancer therapies [81,82] (Figure 3). UPR may also lead to the up-regulation of several PDI family members, which promote cell death. For instance, upregulation of PDIA3 and PDIA19 in neuroectodernal tumors results in the induction of cell death under ER stress [83]. Also, inhibition of PDI sensitizes neuroectodermal cells to ER stress-induced apoptosis, thus demonstrating the vital role played by PDI during ER stress [84,85]. Similarly, dysregulation of PDI expression and activity has been observed in other cancers [86]. PDI inhibition was also found to sensitize hepatocellular carcinoma cells to apoptosis upon the induction of ER stress [87]. Hence, PDI may offer a potential novel target for sensitizing tumor cells to therapy, and therefore it is critical to better understand the specific role played by PDI in cancer progression and the cancer types where this pathway is activated. 

UPR activation serves as a survival strategy for the transformed cells, as cancer usually arises and progresses in a stressful microenvironment. Recently, it has been established that in-vivo activation of UPR is critical for cancer development. Activation of UPR causes transient attenuation of protein synthesis, increased capacity for protein trafficking through ER, proper folding of the proteins, and augmented protein degradation through ER-associated degradation (ERAD) and autophagy. Failure of cells to respond through these adaptive mechanisms undergoes cell death. Therefore, depending on the context, UPR activation contributes to enhanced survival and also induces apoptosis in cancer cells [71]. UPR pathways in mammalian cells consist of three main signaling cascades that are initiated by three primarily ER-localized protein stress markers—namely PKR-like ER kinase, IRE1α (inositol-requiring enzyme 1 alpha), and ATF6 (activating transcription factor 6) [71]. Under physiological circumstances, the luminal domains of PERK and ATF6 are bound to BiP (binding immunoglobulin protein), the ER resident chaperone, and remain inactive [88]. Upon proteotoxic stress, BiP is released from these complexes to enable proper folding of misfolded proteins [89,90,91]. 

BiP has been reported to be overexpressed in several cancers [92,93]. It is a chaperone that enhances cancer cell adaptation to hypoxic microenvironments and confers resistance against anti-cancer therapy [94,95]. In several cancer types, BiP regulates cell proliferation, invasion, apoptosis, inflammation, and immunity [96]. Additionally, BiP has been shown to be involved in angiogenesis, metastasis, and tumorigenesis [89,97,98]. In human cancers including breast, liver, gastric, prostate, and colon, enhanced BiP levels have been correlated with higher pathologic grade, recurrence risk, and poor patient survival [89]. In human breast cancer cells, Grp78 interacts physically and functionally with BIK and inhibits apoptosis mediated by BIK. [99]. It has also been observed that increased expression of BiP decreases sensitivity of glioma cells to etoposide and cisplatin [100]. Thus, BiP is as an effective biomarker indicating aggressive behavior and poor prognosis in cancer [101,102,103,104]. 

The type I transmembrane serine/threonine kinase PERK is enriched at mitochondria associated ER membranes (MAMs) and has kinase activity in the cytosol [105]. Under basal conditions, HSP90 binds to cytoplasmic domain of PERK while BiP binds to the ER luminal domain to stabilize and prevent its activation [106]. Upon ER-stress, BiP binds to misfolded/unfolded proteins, which facilitates the release of PERK, resulting in homodimerization followed by autophosphorylation and the activation of PERK [107,108]. Activated PERK phosphorylates eIF2α at the serine51 residue to attenuate translation initiation [109]. This transitory inhibition of protein synthesis possibly promotes polysome disassembly to increase the number of ribosomes available for binding to newly transcribed mRNAs encoding UPR adaptive functions. In contrast, PERK-dependent phosphorylation of eIF2α upregulates genes that promote amino acid sufficiency and redox homeostasis, thereby promoting cell growth. Phosphorylated eIF2α increases the translation of a number of mRNAs, including those encoding ATF4, ATF5, and amino acid transporters [110,111]. ATF4 translocates into the nucleus to activate UPR genes via binding to amino acid starvation response element (AARE) in genes that are important for antioxidant response, amino acid biosynthesis, and transport, thus promoting cell survival [110]. Under chronic stress, constitutive PERK-mediated phosphorylation of eIF2α can also lead to apoptosis. Hence, it is understood that depending on the severity of stress, activation of PERK promotes both adaptive and apoptotic responses [112,113,114]. In colorectal carcinoma, PERK signaling is crucial for the adaptation of cells to hypoxic stress [115], and activation of the PERK-eIF2α-ATF4 pathway is critical for promoting tumor dormancy, which contributes to chemoresistance in human epidermoid carcinoma cells [116]. It has also been reported by Diane et al. that the PERK-eIF2α-ATF4 pathway confers a survival advantage for hypoxic cells in the tumor mass [117].

Another branch of the UPR pathway that contributes to tumor progression is activated by the type I transmembrane protein IRE1α. IRE1α has a cytosolic serine/threonine kinase domain, which binds both HSP90 and HSP72 while maintaining its stability. BiP binds to the luminal domain of IRE1α to prevent dimerization [106,118]. Upon ER-stress, the accumulation of unfolded proteins in the ER stimulates the release of IRE1α, which oligomerizes and undergoes autophosphorylation, leading to the activation of its kinase and endoribonuclease activities [107,108]. More recently, mammalian IRE1 has been shown to bind peptides and unfolded proteins directly, similar to yeast, resulting in the activation of IRE1 α. Activated IRE1α cleaves XBP1 mRNA to initiate the removal of a 26-base intron in the cytoplasm to produce a translational frameshift that generates a transcriptionally active form of XBP1 (sXBP1) that translocates into the nucleus and binds to promoters of several genes involved in UPR and ERAD [119,120]. Romero–Ramerez et al. showed that hypoxia activates the IRE1–XBP1 arm of UPR, and inhibition of XBP1 inhibits tumor growth [121], which is consistent with other studies suggesting that the loss of XBP1 inhibits tumor growth [122]. Furthermore, hypoxia increases the levels of sXBP1 mRNA and protein [121,123]. Thus, XBP1 acts as an essential survival factor for hypoxic stress and tumor growth [121]. Elevated levels of XBP1 have been observed in many human cancers including breast, hepatocellular, and pancreatic [93,124,125]. IRE1α, on the other hand, promotes apoptosis by stimulating the downstream activation of JNK and p38 MAPK. Apoptosis-inducing substrates of JNK are Bcl-2 and Bim, whereas p38 MAPK activates the transcription factor CHOP [C/EBP homologous protein, also known as growth arrest- and DNA damage-inducible gene 153 (GADD153)], which increases the expression of Bim and DR5 while decreasing the expression of Bcl-2. Acute ER stress activates IRE1α, whereas IRE1α activation is mostly attenuated upon chronic ER stress [126,127,128] through undefined mechanisms. 

ATF6 (activating transcription factor 6) is a type II transmembrane protein that is also a leucine zipper (bZIP) domain containing a transcription factor dependent on cyclic AMP. Under normal conditions, ATF6 is retained and stabilized in the ER through its interaction with BiP. Upon accumulation of misfolded proteins, ATF6 is released from BiP and traffics to the Golgi apparatus, where it undergoes regulated membrane proteolysis by S1P and S2P proteases (also known as MBTPS1 and MBTPS2) [129] to generate an active transcription factor. Cleaved ATF6α mediates the UPR by increasing the transcription of genes that increase ER capacity and the expression of XBP1 [130,131]. To date, no substantial evidence supports the role of ATF6α in ER stress-induced apoptosis. The gene encoding BiP is a transcriptional target of ATF6α and is reported to serve as a marker of malignancy [71]. Upon induction of ER stress, unfolded/misfolded proteins bind to BiP, which results in activation of ATF6α, which in turn ameliorates ER stress [132].

All of the above described arms of the UPR are found to be implicated in different tumor types at different stages of tumor progression. For instance, in the case of hepatocellular carcinoma (HCC), IRE1α-XBP1 signaling was found to be important during the initiation of tumor growth, while once the tumor is established, PERK activation is required [133]. In the context of colorectal carcinoma, PERK signaling was found to be crucial in the adaptation of cancer cells to hypoxic stress, whereas in squamous cell carcinoma, PERK was shown to promote dormancy under adverse microenvironmental conditions [115,116]. In prostate cancer, all three UPR signaling pathways were found to be co-activated and concurrently involved in the malignant progression [134]. 

## 6. Autophagy: A Cellular Stress Response Regulated by HIF-1

During UPR, autophagy sustains cell survival by re-establishing ER homeostasis by digesting the misfolded/unfolded ER proteins. Upon ER-stress, autophagy is stimulated by the IRE1-XBP1 and PERK-eIF2α arms of the UPR [135]. Active IRE1 activates JNK by recruiting TRAF2 (tumor necrosis factor receptor-associated factor 2) and ASK1 (apoptosis signal-regulating kinase). In turn, JNK phosphorylates BCL-2 and BCL-XL, the two autophagy inhibitor proteins, which then dissociate from Beclin 1 [136]. PERK signaling also induces autophagy by activating the expression of ATF4 and CHOP, which drive the expression of the autophagy protein ATG12. ATF4 in combination with CHOP also regulates the expression of TRB3, which inhibits AKT and mTOR, thus inducing autophagy. Calcium released from the ER also activates enzymes such as DAPK (death-associated protein kinase), PKCθ (protein kinase Cθ), or AMPK, which can positively regulate autophagy [135]. Autophagy can also induce apoptosis under ER stress conditions; for example, the inhibition of autophagy has been reported to prevent Caspase-8-mediated cell death [137,138]. Autophagy has also been found to degrade anti-apoptotic factors such as IAPs (inhibitor of apoptosis proteins). PERK was found to degrade XIAP [139]. These reports show that UPR and autophagy are closely associated and can regulate both pro-survival and pro-apoptotic mechanisms. However, there are still important questions to be answered regarding how UPR-induced autophagy and apoptosis are linked. 

## 7. Conclusions

ER stress is critical for the induction of pro-survival mechanisms through the UPR, which allows the adaption to a stressful microenvironment. Recent studies have shown that hypoxia activates the UPR as a mechanism of tumor cell adaptation to low oxygen availability, promoting tumor growth and increased resistance to chemotherapy and radiotherapy. This provides an important mechanism by which hypoxia drives pro-tumorigenic changes and tumor progression. A deeper understanding of the role of hypoxia in inducing ER stress and UPR and the interplay between the two will lead to therapeutic opportunities. Also, it is important to determine the nature of the crosstalk between UPR-induced pro-survival mechanisms (such as autophagy) and cellular death pathways (such as apoptosis). Moreover, UPR markers show different signaling in different cancer types and at different stages, emphasizing the heterogeneity of these responses and the need to consider approaches in specific settings. Finally, it must be considered that, to date, there are no UPR modulators approved for clinical use, thus an important area of study for future research must be approaches for targeting this important stress response pathway to provide novel approaches to cancer therapy.

## Abbreviations

IRE1	Inositol requiring enzyme
PERK	Protein kinase RNA-like endoplasmic reticulum kinase
ATF6	Activating transcription factor 6
JNK	c-Jun N-terminal kinase
TRAF2	TNF receptor-associated factor 2
ASK1	Apoptosis signal-regulating kinase 1
BCL-2	B-cell lymphoma 2
BCL-XL	B-cell lymphoma-extra large
BECN1	Beclin-1
ATF4	Activating transcription factor 4
CHOP	C/EBP homologous protein
TRB3	Tribbles homolog 3
AKT	Protein kinase B (PKB)
mTOR	Mammalian target of rapamycin
